# Effects of Fluoride Exposure on Primary Human Melanocytes from Dark and Light Skin

**DOI:** 10.3390/toxics8040114

**Published:** 2020-12-02

**Authors:** Shilpi Goenka, Sanford R. Simon

**Affiliations:** 1 Department of Biomedical Engineering, Stony Brook University, Stony Brook, NY 11794-5281, USA; sanford.simon@stonybrook.edu; 2 Department of Biochemistry and Cellular Biology, Stony Brook University, Stony Brook, NY 11794-5281, USA; 3 Department of Pathology, Stony Brook University, Stony Brook, NY 11794-5281, USA

**Keywords:** sodium fluoride, neonatal human melanocytes, lightly pigmented, darkly pigmented, cytotoxicity, melanin, tyrosinase, ROS

## Abstract

Fluoride exposure has adverse effects on human health that have been studied in vitro in cell culture systems. Melanocytes are the melanin pigment-producing cells that have a significant role in the regulation of the process of melanogenesis, which provides several health benefits. Melanocytes are present in the oral cavity, skin, brain, lungs, hair, and eyes. However, to date, there has been no study on the effects of fluoride exposure on melanocytes. Hence, in the current study, we have studied the effects of sodium fluoride (NaF) exposure on neonatal human epidermal melanocytes (HEMn) derived from two different skin phototypes, lightly pigmented (LP) and darkly pigmented (DP). We have assessed the impact of a 24 h and 72 h NaF exposure on metabolic activity and membrane integrity of these cells. In addition, we have evaluated whether NaF exposure might have any impact on the physiological functions of melanocytes associated with the production of melanin, which is regulated by activity of the enzyme tyrosinase. We have also assessed if NaF exposure might induce any oxidative stress in LP and DP melanocytes, by evaluation of production of reactive oxygen species (ROS) and measurement of mitochondrial membrane potential (MMP) levels. Our results showed that HEMn-LP cells showed a higher sensitivity to NaF cytotoxicity than HEMn-DP cells, with significant cytotoxicity at concentrations >1 mM, while concentration range 0.25–1 mM were nontoxic and did not lead to oxidative stress, and also did not alter the levels of intracellular melanin or cellular tyrosinase activity, indicating that treatment up to 1 mM NaF is generally safe to melanocytes from both pigmentation phototypes.

## 1. Introduction

Fluoride is the most electronegative trace element in the environment, and humans are exposed to it through drinking water, oral hygiene products, and dietary intake. Although low doses of fluoride possess therapeutic effects against dental caries, exposure to high-dose fluoride has been shown to cause toxicological effects in several cell types and adversely impact human health [1,2]. For example, fluoride has been linked to the development of inflammation and cancer [3]. Another recent study highlighted the toxicity of fluoride on enamel cells in the tooth [4]. The toxicity of fluoride and its mechanisms as well as fluoride resistance by oral pathogens have been reviewed extensively [5,6,7]. Fluorine-containing drugs have been extensively used in the pharmaceutical industry [8]: 18 out of 38 FDA (Food and Drug Administration)-approved drugs contain fluorine [9], in particular, the antifungal drug voriconazole is known to cause skeletal fluorosis and periostitis [10]. Fluoride can cross the placental barrier [11] and maternal exposure to high-dose fluoride during pregnancy has been shown to cause neurocognitive defects in children [12,13]. Epidemiological studies have also validated the adverse effects of fluoride in children, with findings of dental fluorosis and impaired neurodevelopment [14].

Fluoride-releasing dental materials are used for their antibacterial effects and capacity to prevent caries. Most fluoride dentifrices contain fluoride in the form of sodium fluoride (NaF), monofluorophosphate, or stannous fluoride (SnF_2_) [15] as active ingredients. Glass ionomer cements (GIC), a class of dental materials [16], can release higher amounts of fluoride in the first 24 h, termed ‘burst release‘ [17], and can be recharged by the application of topical fluoride varnishes. This converts them to a fluoride reservoir, capable of a sustained release of fluoride over time [18,19]. It has been reported that the amount of toothpaste used by children is usually higher than the recommended pea-size (0.25 g), leading to an excessive intake of fluoride [20], and this is further compounded by children’s habit of swallowing toothpaste during brushing [21]. The differential sensitivity of children and adults to chemical exposures has been previously documented [22]. Neonates and infants are amongst the group at the highest risk for chemical exposure to high-dose fluoride, especially since they have a higher rate of fluoride retention due to a lower glomerular filtration rate. In addition, their lower antioxidant activities [23] and underdeveloped blood–brain barrier [24] puts them at a higher risk of fluoride-induced adverse effects as compared to adults. Furthermore, the risk is aggravated for infants who are exposed to fluoride in the form of beryllium fluoride complexes (which are more toxic than other beryllium or fluoride salts) from the intake of powdered formulas reconstituted with fluoridated tap water [25]. It has been reported that up to 70–75% of mothers use fluoridated tap water to reconstitute infant powdered formulas [26].

Topical or system application of fluoride has been shown to induce cutaneous reactivity in animal models. For example, a fluoride-containing prophylaxis paste that was implanted subcutaneously in rats elicited cutaneous inflammatory reactions [27] and increased histamine levels when applied topically [28]. On the other hand, few studies have documented the in vitro effects of fluoride on keratinocytes, cells which constitute the skin barrier layer in the epidermis [29,30]. Melanocytes are neural-crest-derived cells present in the skin, oral cavity, hair, brain, eyes, and lungs, and are endowed with the unique capacity to synthesize the polymeric pigment, melanin [31], within their specialized lysosome-related vesicles, melanosomes [32]. Tyrosinase is one of the key rate-limiting enzymes involved in the regulation of melanin synthesis within melanosomes by catalyzing the hydroxylation of monophenols to diphenols and the oxidation to diphenols to Dopaquinone [33,34]. The cytoplasmic projections of a melanocyte, called dendrites, serve a critical role in the export of melanosomes from melanocyte dendrite tips to surrounding keratinocytes [35,36]. The critical function of melanin pigment in toxic metal chelation, immune regulation, combating free-radical and ultraviolet (UV) radiation-induced photodamage, has been established [37,38]. Although the presence of melanin contributes to several biological benefits, the disruption of melanocyte homeostasis can lead to severe consequences, such as premature skin aging, increased risk of skin cancer, and pigmentation disorders [39], which include hyperpigmentation or hypopigmentation disorders [40]. Exposure to certain chemicals and pharmaceutical agents has been shown to cause these pigmentation disorders in melanocytes [41].

To date, the effects of fluoride exposure on melanocytes have never been addressed. As melanocytes are also present in the oral cavity, where they aid in preventing inflammation in gingiva [42], the in vitro assessment of fluoride exposure to melanocytes would be a significant step and provide an estimation of cytotoxic doses at which the cellular functions might be impacted for fluoride-containing dental materials. This is especially relevant as fluoride is released for several months inside the oral cavity and might alter functions of gingival melanocytes or induce oxidative stress, thereby impacting normal homeostasis of these cells. Epidermal melanocytes mimic oral melanocytes due to their similar histology and ultrastructure [42,43], and hence can provide clues to the effects of NaF exposure on melanocytes present in oral cavity. Our primary aim was to test the effects of fluoride exposure on human neonatal melanocytes from two different ethnicities (lightly pigmented, LP, and darkly pigmented, DP) by the assessment of cytotoxicity and melanocyte functions of melanin production and tyrosinase activity. Furthermore, the melanocyte model used by us is representative of both the neonatal skin and the gingiva, which also provided us with a broader application of our research.

## 2. Materials and Methods

### 2.1. Materials

Sodium fluoride (NaF; BioXtra, ≥99%, Cat# S7920) and L-dihydroxyphenylalanine (L-DOPA; ≥98%) were purchased from Sigma-Aldrich (St. Louis, MO, USA). MTS (3-(4,5-dimethylthiazol-2-yl)-5-(3-carboxymethoxyphenyl)-2-(4-sulfophenyl)-2H-tetrazolium salt) reagent was procured from Promega Corporation (Madison, WI, USA). Lactate dehydrogenase (LDH) assay, bicinchoninic acid (BCA) protein assay kit, Dulbecco’s phosphate buffered saline (DPBS), Hank’s balanced salt solution (HBSS), antibiotics penicillin-streptomycin (10,000 U/mL), and TrypLE Express (1×) were procured from Thermo Fisher Scientific (Waltham, MA, USA). Cell-lysis buffer (Cat#: EA-0001) was obtained from Signosis Inc. (Santa Clara, CA, USA). Human melanocyte growth supplement (HMGS) and medium 254 were obtained from Cascade Biologics (OR, USA). 2′,7′-dichlorodihydrofluorescein diacetate (H_2_DCFDA) and Tetramethylrhodamine ethyl ester (TMRE) dyes were procured from Molecular Probes (Invitrogen, CA, USA).

### 2.2. Cell Culture

Human epidermal melanocytes from lightly pigmented neonatal donor (HEMn-LP) and darkly pigmented neonatal donor (HEMn-DP) were procured from Cascade Biologics and were maintained in Medium 254 supplemented with 1% HMGS and 1% penicillin-streptomycin in a humidified atmosphere in 95% air 5% CO_2_ incubator at 37 °C. Both cells were used at ≤16 population doublings in assays.

### 2.3. MTS Cytotoxicity Assay

1 × 10^4^ HEMn-LP or HEMn-DP cells/well were cultured in a 96-well plate for 48 h. NaF was dissolved in sterile deionized water at 1 M stock solution, which was diluted further in culture medium to prepare various concentrations (0.25–6 mM) which were added to cells for a period of 24 and 72 h. At the end of treatments, the culture medium was replaced by 100 µL of medium containing 20 µL of MTS reagent and incubated for 2 h. After this, 100 µL were aliquoted to a new 96-well plate and the absorbance was read at 490 nm using a Versamax™ microplate reader. The cell viabilities were reported as % normalized to untreated control after appropriate blank subtractions. Data was analyzed from three biological replicates each with at least three technical replicates.

### 2.4. LDH Membrane Damage Assay

The release of the soluble cytosolic enzyme, LDH, into the surrounding culture medium upon damage to the cellular membrane is a widely used indicator to evaluate membrane integrity of cells and is a measure of cytotoxicity [44]. HEMn-LP or HEMn-DP cells (1 × 10^4^ cells/well) were seeded in 96-well plates for 48 h followed by treatment with NaF at various concentrations for 24 and 72 h. After the exposure period, 50 µL of culture supernatants was transferred to a new 96-well plate with the addition of 50 µL LDH reaction mixture. The positive control consisted of cells treated with lysis buffer supplied with the kit. The plate was covered and incubated for 30 min at room temperature. Subsequently, the stop solution was added, and the absorbance of the plate was read at 490/680 nm using a microplate reader. The results were expressed as % LDH leakage of positive control. Data was analyzed from three biological replicates each with 2–3 technical replicates.

### 2.5. Melanin Production Assay

HEMn-LP or HEMn-DP cells (4 × 10^4^ cells/well) were cultured in 24-well plates for 48 h followed by the replacement of culture medium with NaF at nontoxic concentrations, and cultures were maintained for 72 h. After the treatments, the cells were harvested, washed in PBS, and 100 µL of 1N NaOH was added, and heated at 70 °C to solubilize melanin. Next, 75 µL of lysates was aliquoted in a 96-well plate and the absorbance was read at 475 nm, while a portion of lysate was used for the BCA assay. The absorbances of melanin were normalized to total protein absorbance and the ratio (representing relative melanin levels) was converted to % of untreated control. Data was analyzed from two biological replicates each with at least two technical replicates.

### 2.6. Intracellular Tyrosinase Activity

HEMn-LP or HEMn-DP cells were cultured at 4 × 10^4^ cells/well in 24-well plates for 48 h followed by the replacement of culture medium containing various concentrations of NaF, and cultures were continued for another 72 h. Subsequently, cells were detached, pelleted, washed in PBS, and lysed. Lysates were centrifuged and aliquoted into a 96-well plate with the addition of 3 mM L-DOPA solution. The formation of dopachrome was monitored at 475 nm over a period of 20 min using the kinetic mode setting in a microplate reader. An aliquot was used to estimate protein levels by BCA. The tyrosinase activity was normalized to the total protein content and reported as % of control. Data was analyzed from two biological replicates each with 2–3 technical replicates.

### 2.7. Intracellular Reactive Oxygen Species (ROS) Levels

The intracellular ROS levels were quantitated by the H_2_DCFDA probe using the method similar to that reported previously, with some modifications [45]. Briefly, HEMn-DP or HEMn-LP cells were cultured in 24-well plates (4 × 10^4^ cells/well) for 48 h followed by treatment with different concentrations of NaF, and cultures were maintained for 72 h. Subsequently, the cells were washed in HBSS, 50 µM of H_2_DCFDA probe solution was added, and the plate was incubated at 37 °C for 20 min. After this step, the wells were washed in HBSS, and lysed (using 0.1% Triton-X in phosphate buffer) for 30 min. The lysates were centrifuged and 100 µL of supernatants were transferred in a 96-well black flat-bottom microplate (Greiner Bio-one, Monroe, NC, USA), and fluorescence of DCF was read at excitation/emission of 485/535 nm using a fluorescence microplate reader (Gemini EM, Molecular Devices). The fluorescence intensity (FI) values were normalized to absorbance of total protein contents and expressed as % of untreated control group. Data was analyzed from two biological replicates each with 2–3 technical replicates.

### 2.8. Intracellular Mitochondrial Membrane Potential (MMP) Levels

MMP was quantitated by the cell-permeable cationic probe, TMRE [46]. This probe accumulates in the mitochondria of cells, and cells with depolarized mitochondria show a reduced mitochondria membrane potential (∆ψ_m_) that can be quantitated using a fluorescence plate reader. Briefly, 4 × 10^4^ HEMn-LP or HEMn-DP cells were cultured in 24-well plates for 48 h followed by treatment with NaF, and cultures continued for another 72 h. Subsequently, the cells were washed in HBSS and 200 nM of TMRE probe was added to the cells and the plate was incubated for 20 min. After this step, the wells were washed in HBSS and lysed, the lysates were centrifuged, and 100 µL of supernatants were aliquoted in a 96-well black plate and the fluorescence was read at excitation/emission wavelength of 530/580 nm. The protein contents were calculated from a portion of supernatant and the FI was normalized to the total protein content and expressed as % of untreated control group, similar to the method used for the ROS assay. Data was analyzed from two biological replicates each with 2–3 technical replicates.

### 2.9. Statistical Analysis

The statistical analysis was conducted using one-way analysis of variance (ANOVA) followed by Dunnett’s or Tukey’s post-hoc test when comparing more than three groups, while student’s t-test was used to compare two groups. The significance was considered at *p* < 0.05 and all the analyses were performed using GraphPad Prism version 8.0.0 for Windows, GraphPad Software (San Diego, CA, USA). All data is reported as Mean ± Standard error of mean (SEM).

## 3. Results

### 3.1. Effects of NaF on Metabolic Activity and Membrane Inegrity of HEMn-LP Cells

**Figure 1:**
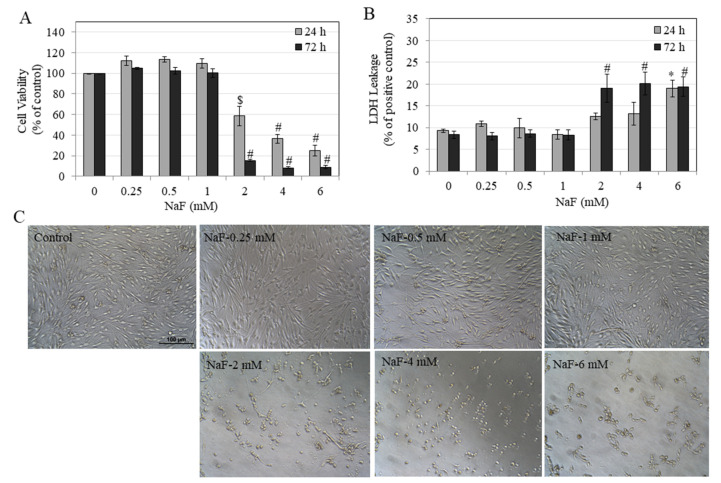
Viability of human epidermal melanocytes lightly-pigmented (HEMn-LP) cells after 24 and 72 h treatment with NaF assessed by (**A**) MTS metabolic activity ($ *p* < 0.001 and # *p* < 0.0001 vs. untreated control) and; (**B**) LDH leakage (* *p* < 0.05 and # *p* < 0.01 vs. untreated control). (**C**) One-way analysis of variance (ANOVA) followed by Dunnett’s post-hoc test was used. Representative images of HEMn-LP cells treated with NaF (0–6 mM) for 72 h, images were taken at 20× magnification. Data for 24 h in (B) is mean ± SEM of at least two independent experiments (*n* = 2) while all other data are mean ± SEM of at least three independent experiments (*n* = 3).

Our results in HEMn-LP cells showed that a 24 h NaF exposure induced a concentration-dependent reduction of the viability of the cells. A significant reduction of 40.78%, 63.08%, and 74.59% was noted at NaF concentrations of 2, 4, and 6 mM, respectively (Figure 1A). More prolonged NaF exposure of 72 h showed a further diminution of cell viability, especially at higher concentration. A significant reduction of 84.64%, 91.68%, and 91.13% was noted at NaF concentrations of 2, 4, and 6 mM, respectively (Figure 1A). Our results showed that under both durations of exposure, NaF over the concentration range 0.25–1 mM was nontoxic to HEMn-LP cells (Figure 1A).

**Figure 2:**
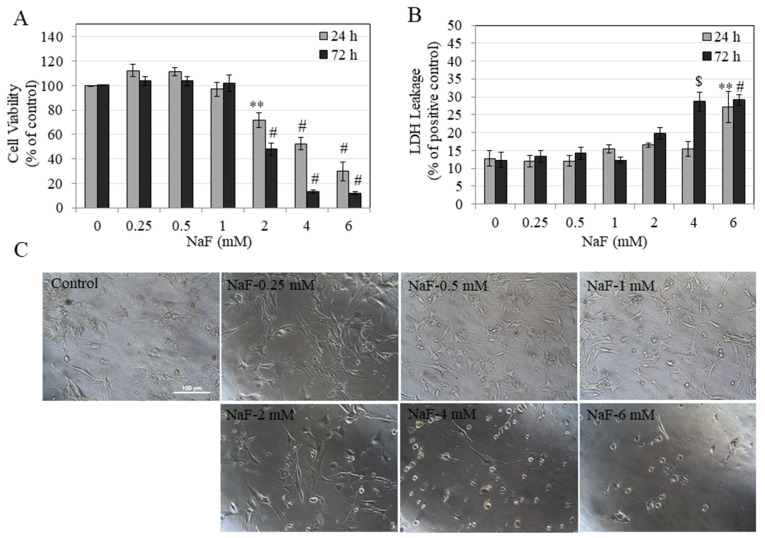
Viability of human epidermal melanocytes darkly-pigmented (HEMn-DP) cells after 24 and 72 h treatment with NaF assessed by (**A**) MTS metabolic activity and (**B**) LDH leakage, ** *p* < 0.01, $ *p* < 0.001, and # *p* < 0.0001 vs. untreated control. One-way ANOVA followed by Dunnett’s post-hoc test was used. (**C**) Representative images of HEMn-DP cells treated with NaF (0–6 mM) for a period of 72 h, images were taken at 20× magnification. All data are mean ± SEM of at least three independent experiments (*n* = 3).

We further evaluated cytotoxicity by assessment of cellular membrane damage, and our results showed that NaF exposure at 24 h induced damage to membranes of HEMn-LP cells, which was significant only at the highest concentration of 6 mM (Figure 1B), while exposure for 72 h significantly damaged cellular membranes at lower concentrations of 2, 4, and 6 mM in a similar manner. Again, over the concentration range of 0.25–1 mM, we observed no signs of membrane damage. We also examined photomicrographs of HEMn-LP cells exposed to NaF for 72 h and confirmed cytotoxicity, and the presence of rounded cell bodies was noted for concentrations of 2, 4, and 6 mM (Figure 2C).

Collectively, these results showed that NaF exposure over a short-term (24 h) and long-term (72 h) induces cytotoxicity only at concentrations exceeding 1 mM, by suppressing cellular metabolic activity, compromising membrane integrity, and inducing necrotic-like changes, which were confirmed by higher LDH release.

### 3.2. Effects of NaF on Metabolic Activity and Membrane Integrity of HEMn-DP Cells

Our results in HEMn-DP cells showed that after a 24 h exposure to NaF, the viability was suppressed in a dose-dependent manner. Significant reductions of viability of 28.11%, 47.12%, and 69.91% were noted at NaF concentrations of 2, 4, and 6 mM, respectively (Figure 2A). NaF exposure for 72 h showed a further suppression of cell viability with significant reductions of 52.66%, 87.14%, and 88.77% at NaF concentrations of 2, 4, and 6 mM, respectively (Figure 2A).

Our results further showed that NaF exposure at 24 h induced damage to membranes of HEMn-DP cells, which was significant only at the highest concentration of 6 mM. Exposure for 72 h induced damage to membranes of DP cells which was significant at 4 and 6 mM NaF (Figure 2B). Evaluation of photomicrographs of cells exposed to NaF for 72 h showed that while cells in the control group had a well-established network of dendritic structures, treatment with NaF at concentrations of 2 mM and higher showed the presence of rounded cell bodies at higher concentrations and loss of dendritic morphology (Figure 2C).

Taken together, these results showed that in HEMn-DP cells, NaF treatment for short-term (24 h) and long-term (72 h) induced cytotoxicity at concentrations >1 mM, similar to our results obtained of HEMn-LP cells.

The half maximal inhibitory concentrations (IC_50_) values were calculated from non-linear regression of dose-response curves of MTS assay for both cells. Our results (Table 1) showed that for both HEMn-LP and HEMn-DP cells, treatment with NaF at longer durations (72 h) induced greater cytotoxicity. The trend was similar for both durations of exposure; however, we noted that HEMn-LP cells exhibited a higher sensitivity to fluoride exposure as compared to HEMn-DP cells for both time points, although comparison by Students’ *t* test showed that these differences failed to reach statistical significance.

### 3.3. Effects of NaF on Intracellular Melanin of HEMn-LP and HEMn-DP Cells

**Figure 3:**
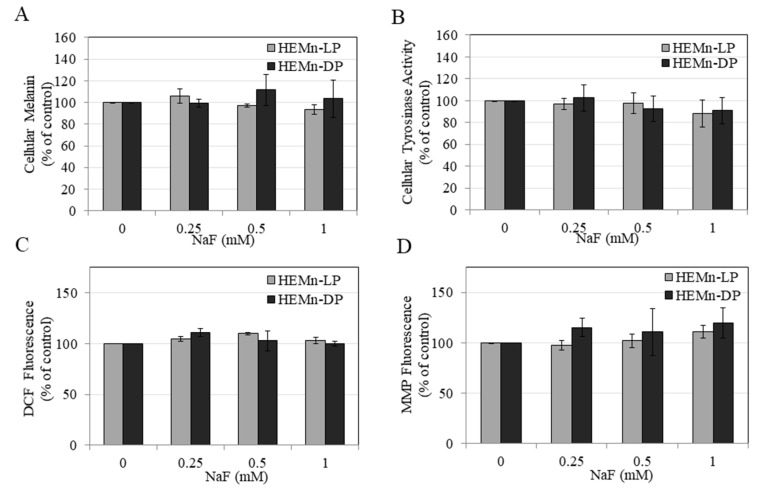
Effects of 72 h treatment with NaF in HEMn-DP and HEMn-LP cells on (**A**) melanin production levels, (**B**) cellular tyrosinase activity, (**C**) cellular reactive oxygen species (ROS) generation, and (**D**) cellular mitochondrial membrane potential (MMP) levels. One-way ANOVA with Tukey’s test, *p* > 0.05 for all groups. All data are mean ± SEM of at least two independent experiments (*n* = 2).

As our results in HEMn-LP and HEMn-DP cells showed that treatment with NaF over concentration range 0.25–1 mM was nontoxic, we have selected these concentrations to evaluate their effects on functions of melanocytes. Our results showed that treatment with NaF over concentration range 0.25–1 mM did not alter the levels of intracellular melanin in both cell types as compared to untreated control (Figure 3A), indicating that NaF exposure does not impact cellular melanin production in melanocytes.

### 3.4. Effects of NaF on Intracellular Tyrosinase Activity in HEMn-LP and HEMn-DP Cells

Treatment with NaF over concentration range 0.25–1 mM did not affect the activity of the enzyme tyrosinase in melanocyte lysates from both donors as compared to untreated control (Figure 3B), indicating that NaF exposure does not affect cellular tyrosinase activity in melanocytes.

### 3.5. Effects of NaF on Intracellular ROS Generation and MMP Levels in HEMn-LP and HEMn-DP Cells

Our results showed that treatment with NaF over concentration range 0.25–1 mM did not affect the ROS levels in melanocytes from both donors as compared to untreated control (Figure 3C) as well as MMP levels (Figure 3D), indicating that NaF exposure does not induce oxidative stress by ROS generation in melanocytes. Collectively, these results highlight that exposure to NaF at nontoxic concentrations does not induce oxidative stress by ROS generation or impair mitochondrial integrity in melanocytes.

## 4. Discussion

In this study, treatment of HEMn cells isolated from neonatal donors differing in skin phototypes showed that concentrations of NaF >1 mM resulted in cytotoxicity characterized by diminished cellular viability, damage to cellular membrane integrity, and loss of the dendritic network of melanocytes. Our results of enhanced LDH leakage by high-fluoride concentrations in both cells are indicative of necrotic cell death which occurs upon rupture of cellular membrane, releasing the LDH enzyme in the extracellular milieu, as reported previously [47]. The adverse effects of reduced cellular metabolism and impaired cellular functions by high-dose fluoride have been shown to be dependent on the cell type, the exposure period, and concentrations of fluoride [48]. Our results of nontoxicity at concentrations <1 mM are in accordance to results of a study which was conducted with gingival fibroblasts, where the authors reported that lower concentrations of 0.5 and 1 mM were nontoxic after 24 h exposure to NaF, with a reduction in cell viability only at a higher concentration of 1.5 mM [49]. A previous study reported that the IC_50_ values for a 24 h NaF exposure to HaCaT cells was 6 mM [29]. Based on our results of IC_50_ values for a 24 h NaF exposure to melanocytes, it can be deduced that HEMn-LP cells are 1.94-fold more sensitive and HEMn-DP cells are 1.51-fold more sensitive than HaCaT cells to fluoride toxicity. Previous studies have documented that the amount of melanin contained within HEMn-DP cells is almost twice that of HEMn-LP cells [50,51]. Our results of reduced sensitivity to cytotoxic concentrations of NaF in HEMn-DP cells might be attributable to the higher melanogenic activity of HEMn-DP cells as compared to HEMn-LP cells. In addition, these differences might also be caused by the presence and ratio of eumelanin/pheomelanin, which is known to differ between light and dark skin phototypes [52]. Alternatively, since HEMn-DP cells have a slower proliferative rate as compared to HEMn-LP cells in culture, the higher sensitivity of LP cells might also be due to faster proliferation. Different sensitivities between cells of the same tissue type has also been reported previously in the case of gingival fibroblasts. Tsutomu et al. reported a higher sensitivity of human fetal gingival fibroblasts as compared to young adult or adult gingival fibroblasts to NaF. The authors attributed these effects to the increased doubling time and higher proliferative capacity of fetal cells as compared to adult cells. Furthermore, the authors reported that a concentration of 1.58 mM completely inhibited cell growth in young adult or adult gingival cells, while similar cell-growth inhibition was obtained in fetal cells at a lower concentration of 1.05 mM [53].

A previous study assessed the exposure of NaF on human hair follicle cells in vitro and described significantly reduced cell growth at NaF concentrations of 1 and 10 mM, and reported ultrastructural images of hair follicles containing melanocytes, although the authors did not assess cytotoxicity to melanocytes [54]. Due to the lack of published reports on the effects of fluoride exposure on melanocytes, we cannot directly compare our results, although a few previous studies [55,56] that were conducted on neural cells bear some similarity to our results of melanocyte cytotoxicity, including a loss of dendritic network at higher concentrations of NaF. High doses of fluoride were shown to impair neurite formation and induce apoptosis in hippocampal neurons from mice [55], while another study documented the loss of axon spines and cytoskeleton alterations in Neuro-2A cells treated with NaF over a concentration range of 2–6 mM [56]. Moreover, a previous study conducted on rats had reported loss of dendritic network of neurons in the brain after chronic exposure to fluoride [57]. Fluoride has been shown to inhibit the activity of various enzymes at millimolar concentrations [58] and a previous study showed that fluoride ions suppressed activity of the soluble tyrosinase enzyme preparation from mushrooms in a cell-free assay, although the authors tested only a single high concentration of 380 mM NaF [59]. The results of this cell-free study cannot be applied to our study since our experiments on tyrosinase activity in cellular assays were conducted with much lower NaF concentrations, in the range of 0.25–1 mM, and our results showed no changes in enzymatic activity. Reactive oxygen species (ROS) include chemically reactive radicals that are normally produced during mitochondrial oxidative metabolism in cells, but their excessive production can cause oxidative stress [60]. Although fluoride has previously been shown to lead to production of ROS and cause mitochondrial dysfunction [61], our results in this study showed that at nontoxic concentrations, fluoride did not impair the ROS generation or mitochondrial integrity of both HEMn-LP and HEMn-DP cells.

The concentration of fluoride has been reported to be variable across different sites in the oral mucosal cavity [62]. For example, a varnish containing 5% NaF (as reported on the label) is 2.26% fluoride by weight, which is equivalent to 22,600 ppm fluoride. After the application of fluoride containing varnish or toothpastes, the reported concentration of fluoride drops drastically within minutes of brushing. For example, it has been reported that the typical salivary fluoride concentrations 1 h after brushing with a high fluoride toothpaste (5000 ppm) and a low fluoride toothpaste (1450 ppm) were 3.21 ppm and 0.90 ppm, respectively [63]. Fluoride released from NaF varnish has been reported to reach much higher concentrations, in the range of 15–25 ppm, during 12 h contact [64], and is known to remain in contact with the tooth for longer times (6–12 h), as compared to mouthrinse or dentifrice. Drinking water contains fluoride in the range of 0.7–1.5 ppm as recommended by the US Public Health Service. One of the limitations of the current study is the in vivo relevance of the concentrations of NaF used in this study since the plasma concentration of fluoride in healthy humans has been reported to be in the range 0.4–3 µM, and in the case of high-dose fluoride exposure, the concentrations are increased by 20-fold [65], which are still far below the concentration ranges tested in the current study. Despite this limitation, our results might be relevant in the case of fluoride exposure from dental products where salivary fluoride concentrations can reach higher levels (up to 25 ppm) based on aforementioned studies. It should be noted that the concentration of 1 mM NaF (42 ppm NaF), which was the threshold concentration after which cytotoxicity was observed in melanocyte cultures in our study, is equivalent to 19 ppm fluoride. The concomitant use of fluoride-containing beverages with the use of high-fluoride-containing varnishes, might have an additive effect in further raising the fluoride concentrations. Polyvalent metal ion-containing fluoride sources such as titanium tetrafluoride (TiF_4_) have shown higher efficacy in combating caries and dental erosion than NaF [66]. In a recent study, the authors compared NaF with TiF_4_, and reported differential cytotoxicity profiles of both fluorides to gingival fibroblasts [67]. Future studies to compare the effects of exposure of TiF_4_ and NaF and testing them as a varnish formulation with melanocytes would provide further significant insights. A limitation of this study is that we have used melanocytes from a single donor from both pigmentation types (LP and DP), hence further studies to test the results with cells from multiple donors of the same phototype might provide a more rigorous dataset.

Within the limitations of the current in vitro study, we can conclude that NaF at concentrations >1 mM adversely impacted cellular metabolic status and induced cytotoxicity, although NaF exposure over a period of 72 h failed to elicit cytotoxicity and alterations of cellular functions at concentrations <1 mM (which are well-below the IC_50_ values). We did not evaluate the effects of concentrations >1 mM on melanocyte functions due to cytotoxicity, as we focused on nontoxic concentrations of NaF to study the impact on cellular functions. Although our results show a negligible impact of a short-term exposure of fluoride on functions of melanocytes, whether a chronic exposure of several weeks might alter functions of melanocytes remains to be elucidated. Furthermore, it should be emphasized that in vivo, melanocytes are in close contact with keratinocytes, which regulate melanin synthesis in melanosomes and export of melanosomes from melanocytes [68,69]. A previous study conducted with osteoblasts documented that fluoride at concentrations of 0.5 and 1 mM impacted gap-junction intercellular communication (GJIC) and attenuated the levels of the key GJIC protein, connexin 43 (Cx43) [70]. As it has been reported that melanocytes are also capable of heterocellular GJIC by these connexin proteins [71], whether fluoride might similarly impact the intracellular communication between these cells or might affect melanosome export would also be worthy of future investigation. Fluoride has been known to selectively inhibit glycolysis of microbes of the gingival plaque that are known to be cariogenic and will therefore inhibit the anerobic bacteria that cause tooth decay, but melanocytes, like other mammalian cells, have functional mitochondria and can deal with a certain extent of inhibition of the glycolytic pathway. We report the novel finding in this study that melanocytes are relatively resistant to fluoride exposure up to concentrations of 1 mM. Our findings have significant implications for future investigation, which are especially pertinent to the field of dental research. As HEMn-LP and HEMn-DP cells have been shown to exhibit a differential expression of cytokines under stimulation by bacterial lipopolysaccharides [72], future studies to test whether fluoride might affect cytokine secretion in melanocytes stimulated with lipopolysaccharides of bacterial plaque remain to be clarified. In addition, future research incorporating keratinocytes and evaluation of their role in modulating response of melanocytes to fluoride exposure would provide a more physiological context which can mimic the in vivo cell-cell interactions. For instance, the use of gingival keratinocytes with melanocytes in a coculture model exposed to fluoride over longer durations of exposure would be an interesting follow-up study.

## 5. Conclusions

Our research findings on the effects of fluoride on melanocytes have significant implications and might help in providing guidelines for the younger population (infants and children) that are more vulnerable to fluoride toxicity by ingestion (in the form of infant powdered formulas reconstituted with fluoridated water and swallowed toothpastes). In addition, our findings are also applicable for the identification of cytotoxicity of fluoride-containing dental products to oral melanocytes since the cell model used in this study closely mimics oral melanocytes, which, if extrapolated to the in vivo situation, is representative of oral melanocytes from healthy gingiva and the pigmented gingiva that is typical of smokers. Lastly, our results also provide novel data regarding the effects of fluoride compared across different ethnicities which might be applicable to diverse populations.

## Figures and Tables

**Table 1:** Half maximal inhibitory concentration (IC_50_) values of NaF cytotoxicity assessed by MTS assay at 24 and 72 h in HEMn-LP and HEMn-DP cells. Values are mean ± SEM from three independent experiments (*n* = 3).

Cell Type	IC_50_, 24 h (mM)	IC_50_, 72 h (mM)
HEMn-LP	3.09 ± 0.45	1.68 ± 0.09
HEMn-DP	3.97 ± 0.61	2.05 ± 0.13
